# Congenital Smooth Muscle Hamartoma Obscuring the Cornea

**DOI:** 10.1155/2021/6692229

**Published:** 2021-05-08

**Authors:** Andrew Bean, Abdullah Al-Bouri, Geoffrey Bradford, Thomas Mauger

**Affiliations:** ^1^Department of Ophthalmology and Visual Sciences, West Virginia University School of Medicine, 1 Medical Center Drive, P.O. Box 9193, Morgantown, WV 26506, USA; ^2^Department of Pathology, Anatomy and Laboratory Medicine, West Virginia University School of Medicine, WVU Department of Pathology, Room 2187 HSN, P.O. Box 9293, Morgantown, WV 26056, USA

## Abstract

Congenital smooth muscle hamartoma is a benign proliferation of smooth muscle that most commonly presents in the lumbosacral area or proximal extremities. Although exceedingly rare, congenital smooth muscle hamartomas have been reported to occur in ocular structures such as the brow, eyelid, and conjunctival fornix. This case describes an atypical congenital bulbar lesion in a newborn male which obscured the cornea. The lesion, which appeared to originate from the bulbar conjunctiva and/or the limbus, was excised at 5 months of age. Pathologic evaluation was consistent with congenital smooth muscle hamartoma. The authors of this report believe it is the first to describe a patient with a congenital smooth muscle hamartoma of the bulbar conjunctiva/limbus.

## 1. Introduction

A congenital epibulbar mass raises suspicion for a variety of conditions ranging from benign to life-threatening. High on the differential is a limbal dermoid, which typically presents as a well-circumscribed yellow-white solid mass involving the conjunctiva, limbus, or cornea [1]. A more rare condition with similar presentation is congenital anterior staphyloma, consisting of an opaque corneal protrusion lined with uveal tissue. Histopathologic evaluation is often helpful in establishing the true etiology of these lesions.

Congenital smooth muscle hamartoma is a benign proliferation of smooth muscle that predominately occurs as a single lesion in the lumbosacral area and proximal extremities. These lesions are sporadic and occur in 1 in 2600 live births, with a slight male predominance. Ocular involvement of congenital smooth muscle hamartoma, though exceedingly rare, has been reported in the eyelid, brow, and conjunctival fornix. The authors believe this report is the first to describe a patient with a congenital smooth muscle hamartoma in the bulbar conjunctival/limbal region.

## 2. Case Report

**Figure 1:**
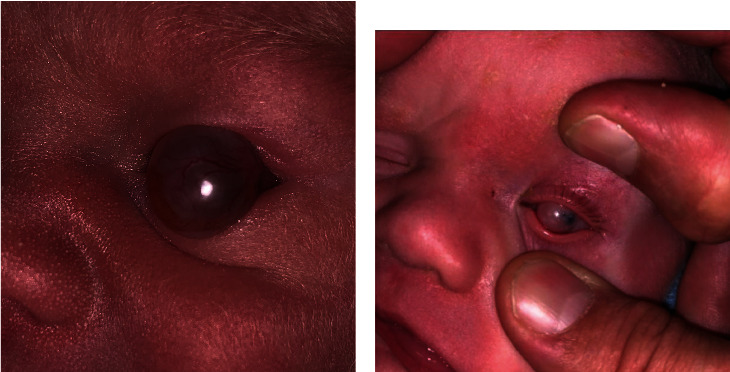
(a) External photograph taken on the day of birth showing the cystic lesion protruding from the ocular surface. (b) External photograph taken at 2 months. Mild regression of protrusion with persistent corneal coverage.

**Figure 2:**
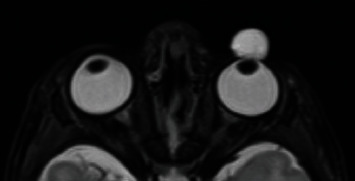
T2-weighted MRI of the orbits, axial view. Unilocular cyst arising from the anterior portion of the left globe.

A newborn male was seen in the hospital for a cyst on his left eye. The cyst had been visualized by prenatal ultrasound at 28 weeks of gestation. The patient was born at 38 weeks and 2 days. There were no complications with delivery; vacuum-assist or forceps were not required. On ophthalmic examination, the cyst was translucent and vascularized. It was 9 mm in size and protruded from the globe (Figure 1(a)). It was attached to the limbus nasally and temporally and to the sclera inferiorly. Magnetic resonance imaging of the brain and orbits with and without contrast reported a unilocular cyst arising from the left globe (Figure 2). No other abnormal findings were noted. The lesion was thought to be an atypical dermoid cyst. Erythromycin ointment was prescribed, and a close follow-up was arranged.

The cyst was noted to have regressed somewhat at the patient's two-month clinic visit, with less protrusion from the globe than initial presentation. It appeared as a firmly attached, pink infratemporal mass (Figure 1(b)). Some peripheral cornea was visible, but the pupil was occluded completely. Little if any potential for useful vision was possible with the lesion in place. After discussion with the patient's parents, the decision was made to proceed with excision of the cyst and cornea graft placement.

The cyst wall was incised superiorly, and dissection was carried inferiorly. The wall of the cyst was completely excised from the underlying deformity and sent for pathology. Removal of the cyst resulted in an evident defect in the cornea, with uvea protruding through. Once dissection of the trephination extended to the inferior temporal area, the iris had to be caught and peeled off the back of the adherent cornea. The lens did not appear to be involved and was retained. The donor cornea was transferred into place and sutured with interrupted 10-0 nylon sutures.

The patient tolerated the procedure well. On postoperative day 1, the graft was in good position and the closure was intact. However, the graft had become diffusely cloudy by two months postoperatively and by 3 months had developed peripheral vascularization. The patient did not seem to have any pain or distress with his left eye. Given the extensive nature of the original lesion and involvement of three-fourths of the limbus, a repeat graft was thought to have a high likelihood of failure.

**Figure 3:**
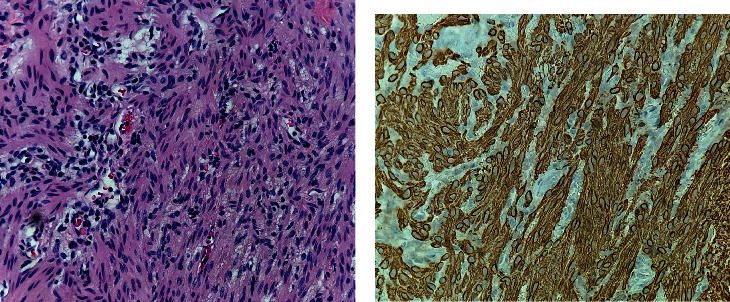
(a) 400x magnification Hematoxylin and Eosin stain showing smooth muscle cell proliferation without atypia. (b) 400x magnification showing diffusely positive smooth muscle actin staining.

Pathologic evaluation of the specimen revealed conjunctival mucosa with benign smooth muscle proliferation, consistent with smooth muscle hamartoma. Hematoxylin and Eosin staining revealed haphazardly arranged fascicles of smooth muscle with characteristic elongated, spindle-shaped to blunt-ended nuclei (Figure 3(a)). Smooth muscle actin stain was diffusely positive (Figure 3(b)). The lesion lacked dense fibrous tissue or dermal elements characteristic of dermoid. Upon subsequent follow-up, the patient's parents gave written informed consent for the publication of his case, including the use of external photographs.

## 3. Discussion

Congenital smooth muscle hamartomas of the periorbita are thought to arise from the vascular smooth muscle or from the smooth muscle in the eyelid retractor complexes. Though rare, there are several cases of congenital smooth muscle hamartomas of ocular structures described in the literature.

Roper et al. described a 2-year-old African American male with a three-month history of a left lower eyelid mass and no other ocular abnormalities. When excisional biopsy was performed, areas of the lesion were noted to be adherent to the inferior border of the tarsus and tarsal conjunctiva. Histologic evaluation was consistent with the diagnosis of congenital smooth muscle hamartoma [2].

Another report by Mora et al. reviewed the case of a healthy five-year-old boy from Mexico City who presented for the evaluation of a cystic right lesion of the right eye that had been present since birth. The lesion, which appeared reddish-pink, was determined to emerge from the upper eyelid conjunctiva. Excisional biopsy and subsequent pathologic studies revealed findings consistent with congenital smooth muscle hamartoma of the tarsal conjunctiva. [3]

Finally, Johnson and Jacobs published a case series of six cases of congenital smooth muscle hamartomas. One case described an 11-year-old female with a pinkish-tan lesion involving her right eyelid and brow. In addition to positive histopathologic findings, the lesion also presented with a positive pseudo-Darier's sign—defined as a transient piloerection and induration of the lesion induced by rubbing. This sign helps clinically distinguish congenital smooth muscle hamartoma from congenital hairy nevus. [4, 5]

Literature search on PubMed for ocular and periocular congenital smooth muscle hamartomas yielded the aforementioned cases. To our knowledge, this is the first reported case of a congenital smooth muscle hamartoma originating in the bulbar conjunctival/limbal tissue. It is possible that our patient's congenital smooth muscle hamartoma arose from the smooth muscle cells the limbal vascular endothelium. Moving forward, congenital smooth muscle hamartoma should be added to the differential in a patient presenting with a cystic corneal or perilimbal lesion.

## Data Availability

Data sharing is not applicable to this article as no new data were created or analyzed in this study.
